# Non-severe burn injury increases cancer incidence in mice and has long-term impacts on the activation and function of T cells

**DOI:** 10.1093/burnst/tkac016

**Published:** 2022-04-29

**Authors:** Lucy W Barrett, Vanessa S Fear, Bree Foley, Katherine Audsley, Samantha Barnes, Hannah Newnes, Alison McDonnell, Fiona M Wood, Mark W Fear, Jason Waithman

**Affiliations:** 1 Burn Injury Research Unit, School of Biomedical Sciences, University of Western Australia, Crawley, WA, 6009, Australia; 2 Telethon Kids Institute, University of Western Australia, Northern Entrance, Perth Children’s Hospital, 15 Hospital Ave, Nedlands, WA, 6009, Australia; 3 Fiona Wood Foundation, Fiona Stanley Hospital, MNH (B), Main Hospital, CD 15, Level 4, Burns Unit, 102-118 Murdoch Drive, Murdoch, WA, 6150, Australia; 4 Burns Service of Western Australia, WA Department of Health, Nedlands, WA, 6009, Australia

**Keywords:** Immunology, Surgery, Burn injury, T cells, Immune dysfunction, Cancer

## Abstract

**Background:**

Recent evidence suggests that burn patients are at increased risk of hospital admission for infection, mental health conditions, cardiovascular disease and cancer for many years after discharge for the burn injury itself. Burn injury has also been shown to induce sustained immune system dysfunction. This change to immune function may contribute to the increased risk of chronic disease observed. However, the mechanisms that disrupt long-term immune function in response to burn trauma, and their link to long-term morbidity, remain unknown. In this study we investigated changes to immune function after burn injury using a murine model of non-severe injury.

**Methods:**

An established mouse model of non-severe burn injury (full thickness burn equivalent to 8% total body surface area) was used in combination with an orthotopic model of B16 melanoma to investigate the link between burns and cancer. Considering that CD8^+^ T cells are important drivers of effective tumour suppression in this model, we also investigated potential dysregulation of this immune population using mouse models of burn injury in combination with herpes simplex virus infection. Flow cytometry was used to detect and quantify cell populations of interest and changes in immune function.

**Results:**

We demonstrate that 4 weeks after a non-severe burn injury, mice were significantly more susceptible to tumour development than controls using an orthotopic model of B16 melanoma. In addition, our results reveal that CD8^+^ T cell expansion, differentiation and memory potential is significantly impaired at 1 month post-burn.

**Conclusions:**

Our data suggests that CD8^+^ T cell-mediated immunity may be dysfunctional for a sustained period after even non-severe burn injury. Further studies in patients to validate these findings may support clinical intervention to restore or protect immunity in patients after burn injury and reduce the increased risk of secondary morbidities observed.

## Graphical Abstract

**Graphical Abstract:**
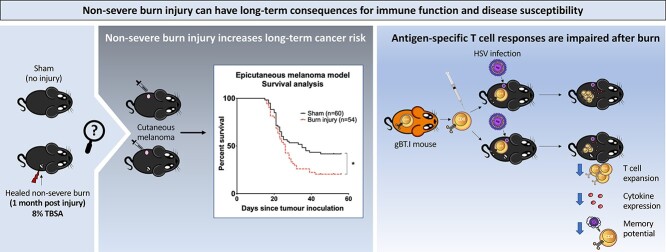

Highlights

- Non-severe burn injury increases the incidence of tumour development in mice, suggesting immune dysfunction is sustained after burn injury.
- Non-severe burn injury has a sustained impact on immune function, characterized by inefficient CD8^+^ T cell responses.
- Potential defects in CD8^+^ T cell immunity may increase susceptibility to disease in burn patients after recovery.

## Background

Significant advances in surgery and post-burn wound care have improved the survival of burn patients over recent years. However, there is growing evidence that burn injury has long-term impacts on patient health long after discharge [1]. In Western Australia, a hospital-linked population study involving >30,000 burn patients showed that burns are associated with an increase in secondary morbidities for many years after discharge from hospital for the burn injury itself. These include musculoskeletal and nervous system disorders [2,3], gastrointestinal and cardiovascular diseases [4,5], influenza and respiratory infections [6] and cancer [7]. In this population study only 3% of patients suffered a severe burn, suggesting that even non-severe burns have a lifelong impact [8,9]. These studies further demonstrated that burn injury sustained by children was associated with an increased risk of age-adjusted mortality of all causes [10], strongly supporting the existence of a sustained and long-term physiological impact of burn trauma on health.

A growing body of evidence suggests that burn injury induces persistent changes to immune function in the paediatric population [1] that may underpin susceptibility to secondary morbidities. A study of severely burned children found that cytokines Interleukin 10 (IL-10), Granulocyte-macrophage-colony stimulating factor (GM-CSF), Tumor necrosis factor alpha (TNF-α), IL-2 and IL-17 were significantly elevated up to 3-years post-injury [11], indicative of a suppressive immune phenotype. A more recent study in non-severe burn paediatric patients [<10% total body surface area (TBSA)] similarly showed sustained increases of TNF-α, IL-2, IL-7 and Interferon gamma (IFN-γ) >3 years post-injury [12], and analysis of peripheral blood mononuclear cells showed significant increases in myeloid dendritic cells, memory CD4^+^ T cells and memory T-regulatory cells, providing evidence of long-term alterations in immune cell populations in paediatric burn patients. While similar studies are lacking in adults, the observation that even non-severe burn injury is associated with significantly increased risk and severity of infectious diseases and respiratory infections (determined by both admission rates and length-of-stay in hospital) over a follow-up period of around 15 years post-burn injury [6,8], suggests that immune dysfunction is also likely occurring in the adult burn population. The increase in respiratory infections observed in these studies was also observed in paediatric patients after burn [13], providing further evidence that burn has similar effects on immune function in adults. However, the mechanisms that underlie immune dysfunction in children and adults may not be the same and will be important to investigate in future studies.

The immune system plays a critical role in cancer prevention, detecting and removing malignant cells prior to tumour progression [14]. In this study, we examined whether the apparent increased risk of cancer observed in burn patients could be recapitulated in preclinical models of non-severe burn injury. We utilized our well-established mouse model of non-severe burn injury in which anaesthetized mice are subject to an 8% TBSA burn on their right hand flank [15], followed by epicutaneous inoculation of B16 melanoma cells on the contralateral flank 1 month later [16]. In this model, disease progression reflects stages seen in patients, with tumours arising in the epidermis, followed by dermal invasion and spontaneous metastatic spread, with disease outcomes dependent on CD8^+^ T cell immunity [17]. In this study, we demonstrate that non-severe burn injury leads to significantly increased incidence of melanoma, consistent with patient data showing increased incidence of cancer in the years after burn injury [7,18]. We also characterized the immune response of CD8^+^ T cells, CD4^+^ T cells, dendritic cells (DCs) and natural killer (NK) cells post-burn using a transgenic herpes simplex virus (HSV) model to understand the functional impacts of non-severe burn injury on the immune response.

## Methods

### Mice

Female C57BL/6 mice (8 weeks old) were purchased from the Animal Resource Centre, Western Australia and housed under pathogen-free conditions with food and water provided *ad libitum*. Female mice were used for this study as female burn patients have increased risk of cancer [18] and mortality post-burn [19]. gBT.I [20] and gDT.II [21] transgenic mice were bred at the Telethon Kids Institute. All studies were approved by the Telethon Kids Institute’s Animal Ethics Committee (AEC, #338) and all experiments were performed in accordance with the National Health and Medical Research Council Australia Code of Practice for the Care and Use of Animals for Scientific Purposes.

### Full-thickness burn procedure

Female C57BL6/J mice (9 weeks old) received a full-thickness 19-mm diameter burn wound as described [15], that equates to ~8% TBSA. Sham injury mice received no surgical treatment but underwent anaesthesia and were shaved on their flank. Animals were administered analgesic (buprenorphine, 0.1 mg/kg) subcutaneously immediately post-injury and at 12 h. Water was instilled with paracetamol (1 mg/ml) for 5 days following surgery.

**Cell lines**

B16-F10 (B16) murine melanoma cells were purchased from the ATCC and routinely passaged and cultured at 70–80% confluency in RPMI medium (Life Technologies) supplemented with 10% FCS (Sigma-Aldrich), 2 mM L-glutamine, 50 μM 2-mercaptoethanol, 100 μg/mL streptomycin and 100 U/mL penicillin (all Life Technologies) (R10 media) at 37°C, 5% CO_2_. A lymphoma mouse cell line that are sensitive to the activity of natural killer cells (YAC) (kindly provided by Dr Jerome Coudert, Lions Eye Institute, Western Australia) were cultured in R10 medium at 37°C, 5% CO_2_.

### Cutaneous melanoma engraftment

Mice were anaesthetized with a mixture of ketamine (100 mg/kg) and xylazil (10 mg/kg) (Troy Laboratories) administered intraperitoneally (10 mL/g body weight).

Refresh®Lacri-Lube® (Allergan) was applied to mouse eyes for the duration of anaesthesia. The flank of the mouse was shaved and depilated with Veet®(Reckitt Benckiser). A small (~2 mm^2^) area of surface skin on the flank was abraded using a MultiPro Dremel with a grindstone attachment on low speed. B16 melanoma cells (10^5^) were washed, resuspended in 10 μL of Matrigel™ (BD), applied to the abraded area and allowed to set. To contain the cells, the abraded site was covered with a piece of Op-site FlexigridTM (Smith and Nephew), then the torsos of the mice were wrapped, first with a soft hypoallergenic MicroporeTM tape (3 M Health Care) and then a second layer of Fixomull Stretch (BSN Healthcare). Mice recovered from anaesthesia on a heating pad and were monitored daily until bandages were removed 5 days post-grafting. Mice with tumours >1000 mm^3^ were euthanised. Tumour-free mice were defined as mice with no palpable masses.

### HSV infection model

Mice were infected with 1 × 10^6^ plaque-forming units of HSV-KOS (HSV-1) following the flank scarification model [22]. This model is technically very similar to the cutaneous melanoma engraftment described above, with two main differences: (1) HSV-1 was applied to the skin instead of B16 cells and (2) bandages were only left on for 2 days and a stronger porous polyethene TransporeTM tape (3 M Health Care) was used for the second layer instead of Fixomull. Previous characterization of this model demonstrated that expansion of HSV-specific T cells peaks at day 5 in the draining lymph nodes and at day 7 in the spleen [23]. Therefore extraction and analysis of splenocytes in this study was conducted at day 7 post-HSV infection.

### Fluorescent activated cell sorting (FACS) analysis and antibodies

Monoclonal antibodies specific to mouse CD3, CD8a, CD4, CD45.1, Vα2, IFN-γ, IL-2, TNF-α, CD19, CD11b, NK1.1, CD62L, CD44, CD27, CD127, granzyme B (GzmB), KLRG1 and T cell transcription factor 1 (TCF1) and Fixable Viability Stain were purchased from BD or eBioscience. Multiparameter analysis was performed on a LSRFortessa (BD) and analysed using FlowJo software (TreeStar, Ashland, OR).

### Preparation of single-cell solutions from mouse spleen and lymph nodes

Spleens and lymph nodes were dissected from mice post mortem, placed in cold Phosphate buffered saline (PBS) then minced through sterile 70 mm wire mesh. Erythrocytes were removed with red blood cell lysis buffer (Thermo Fischer). Single-cell solutions for spleen and lymph nodes were prepared by pipetting cells through a 100 μM cell strainer.

### *Ex vivo* intracellular staining assay for CD4^+^ and CD8^+^ T cells

For CD4 intracellular staining (ICS), MuTu DCs were plated into V-bottomed 96-well plates (4 × 10^4^ cells per well, 100 μL volume) and cultured overnight. The next morning, cells were centrifuged and appropriate wells were pulsed with 50 μL/5 μM gD_287–317_ peptide (Mimotopes) in FCS-free RPMI for 1 h at 37°C. Single-cell preparations of spleen from mice 7 days-post HSV infection were plated into V-bottomed 96-well plates (CD4 ICS: 2 × 10^5^ cells in 150 μL volume for unstimulated and gD-stimulated wells, 1 × 10^5^ for phorbol 12-myristate 13-acetate - a protein kinase C activator (PMA)-stimulated wells; CD8 ICS: 4 × 10^5^ cells in 180 μL for unstimulated and gB wells, 2.5 × 10^5^ for PMA wells). gB_498–505_ peptide (1 μM; Mimotopes) was added to the appropriate gB-stimulated wells, and PMA was added to the appropriate wells in both CD4 and CD8 plates. All wells were made up to a total volume of 200 μL and incubated at 37°C for 1 h. Next, 100 ng/mL brefeldin A (Golgi Plug; BD Biosciences) was added to each well and the cells were incubated for an additional 4 h. Cells were then centrifuged and washed in PBS prior to staining with Fixable Viability Stain 575 V at 1:20,000 (BD Biosciences) for 20 min in the dark at room temperature. Cells were centrifuged and washed in FACS wash prior to extracellular antibody staining for 20 min at 4°C. CD4 plates were stained for CD4, CD45.1 and Vα3.2, CD8 plates were stained for CD8a, CD45.1 and Vα2. Cells were washed and fixed in PBS containing 2% paraformaldehyde and then permeabilized and stained intracellularly for IFN-γ, IL-2 and TNF-α for 20 min at 4°C before being washed and analysed on a LSRFortessa (BD).

### Antigen presentation assay

Antigen presentation by DCs during HSV infection was assessed using a previously described protocol [23]. Briefly, gBT.I cells were incubated with CFSE (2.5 μM) at 37°C for 10 min in PBS/0.1% BSA, washed and resuspended in RPMI. Each mouse received 7.5 × 10^5^ cells intravenously at 2 or 5 days post-HSV infection. Then, 42–60 h later mice were sacrificed and lymph nodes were taken for analysis on a LSRFortessa. As the cells divide the CFSE intensity is halved, giving peaks of different intensities that correlate with the number of cell divisions.

### NK cell activity assay

Single-cell preparations of spleen from mice 7 days-post HSV infection were plated into V-bottomed 96-well plates (5 × 10^5^ cells per well, 100 μL volume). Cells were unstimulated, stimulated with PMA (100 ng/ml) and calcium (0.5 μM) or cultured 2 : 1 with YAC cells, to a final volume of 200 μL. Anti-CD107α was added to each well and incubated for 1 h at 37°C and 5% CO_2_. After 1 h, brefeldin A and monensin (Golgi Stop; BD Biosciences) were added to each well and cells were incubated for 4 h. Cells were then centrifuged and washed in PBS prior to staining with dead cell stain (AF700) for 20 min in the dark at room temperature. Cells were centrifuged and washed in FACS wash prior to extracellular antibody staining for 20 min at 4°C with CD3, CD19, CD11b, CD27 and NK1.1. Cells were washed and fixed in PBS containing 2% paraformaldehyde and then permeabilized and stained intracellularly for IFN-γ for 20 min at 4°C and then analysed on a LSRFortessa.

### Statistical analysis

Statistical analysis was performed using GraphPad Prism 9 (GraphPad Software, Inc., USA). All data are presented as mean ± SD. Survival curves were compared using the Mantel–Cox log-rank test. All data was tested for normality using the Shapiro–Wilk test. For all datasets with normal distribution and comparisons involving two groups only, Welch’s t-test was used. Welch’s t-test was used to compare sham and burn injured groups. For experiments with more than two experimental groups and normal distribution one-way analysis of variance - a collection of statistical models (ANOVA) was performed with Dunnett’s *post hoc* test. For datasets that were non-normally distributed either Mann–Whitney (comparison of two datasets) or Kruskal–Wallis non-parametric tests with Dunn *post hoc* test (more than two datasets) was used. Differences were considered statistically significant at *p* < 0.05.

## Results

### Non-severe burn injury results in increased cancer incidence in a cutaneous model of melanoma

**Figure 1.**
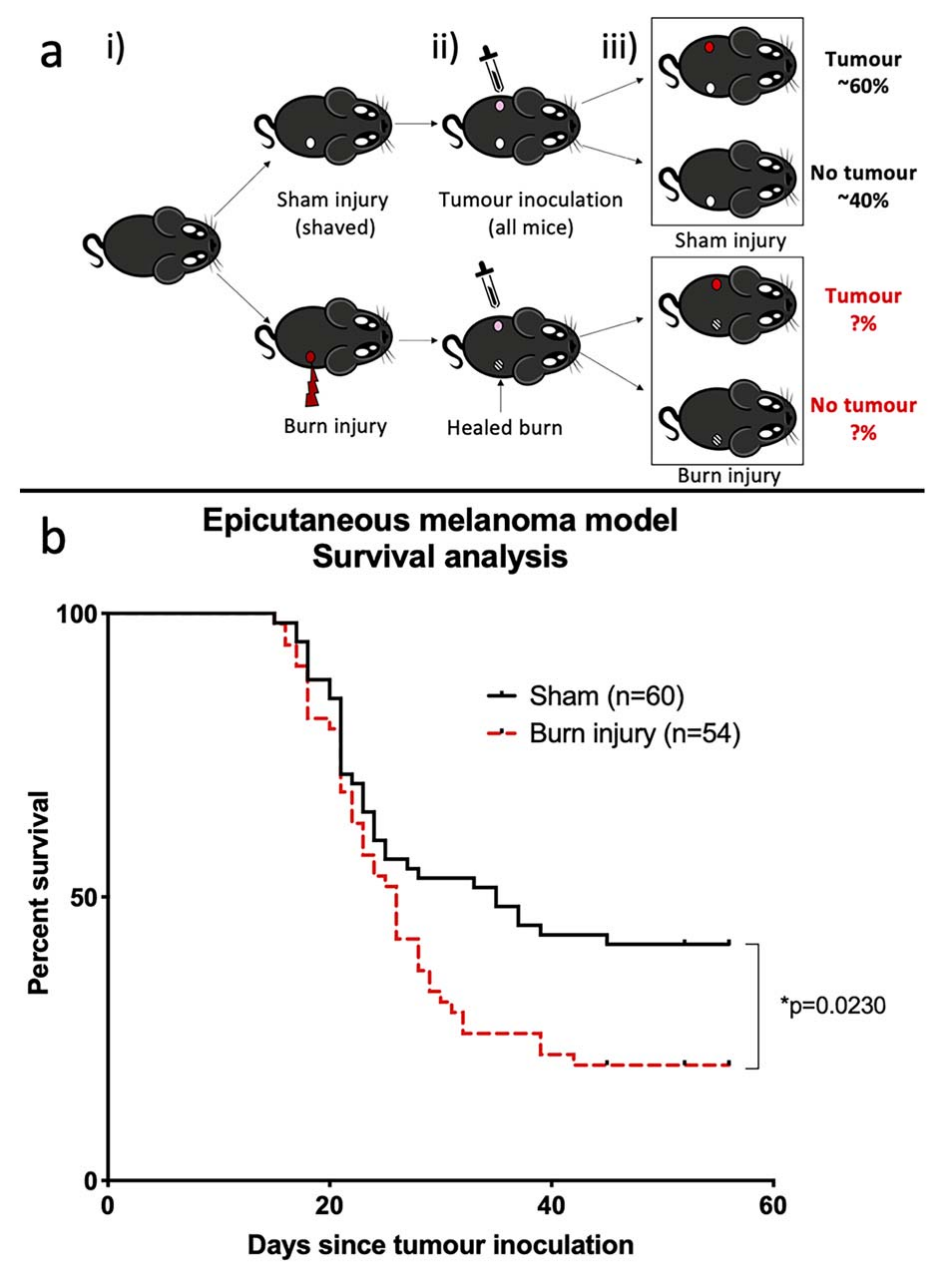
Model of burn injury and cutaneous melanoma. (**a**) Schematic diagram of the experiment. Female C57BL/6 mice were (i) subjected to sham (control) or non-severe burn injury, (ii) epicutaneously inoculated with B16 melanoma cells on the opposite flank at day 28 post-injury and (iii) and monitored over time for tumour development. (**b**) Tumour-free survival in sham (*n* = 60) or burn injured (*n* = 54) mice (combined data from n = 6 experiments). Burn injured mice had a significant increase in mortality, with a median survival of 35 days in sham mice compared to 26 days in burn injured animals (^*^p = 0.023)

We utilized an orthotopic melanoma model 4 weeks after mice were exposed to a non-severe burn injury equivalent to ~8% TBSA. Consistent with published data this led to macroscopic tumour development in the dermis and epidermis in ~60% of sham-injured mice 2–4 weeks post-inoculation (Figure 1). Burn injured mice had significantly decreased survival, with a median survival of 35 days in sham- and 26 days in burn-injured animals (*p* = 0.023). There was significantly increased tumour incidence in the burn injury group (79.63%) compared to controls (58.33%), supporting the existence of a sustained immune defect after non-severe burn. In mice in which tumours did appear, there was no observable difference in tumour morphology or growth rates between the sham and burn groups, indicating there was no difference in tumour characteristics between the two groups (Figure S1, see online supplementary material).

### Antigen-specific CD8^+^ T cell expansion and cytokine production is impaired in the response to HSV infection after burn injury

**Figure 2.**
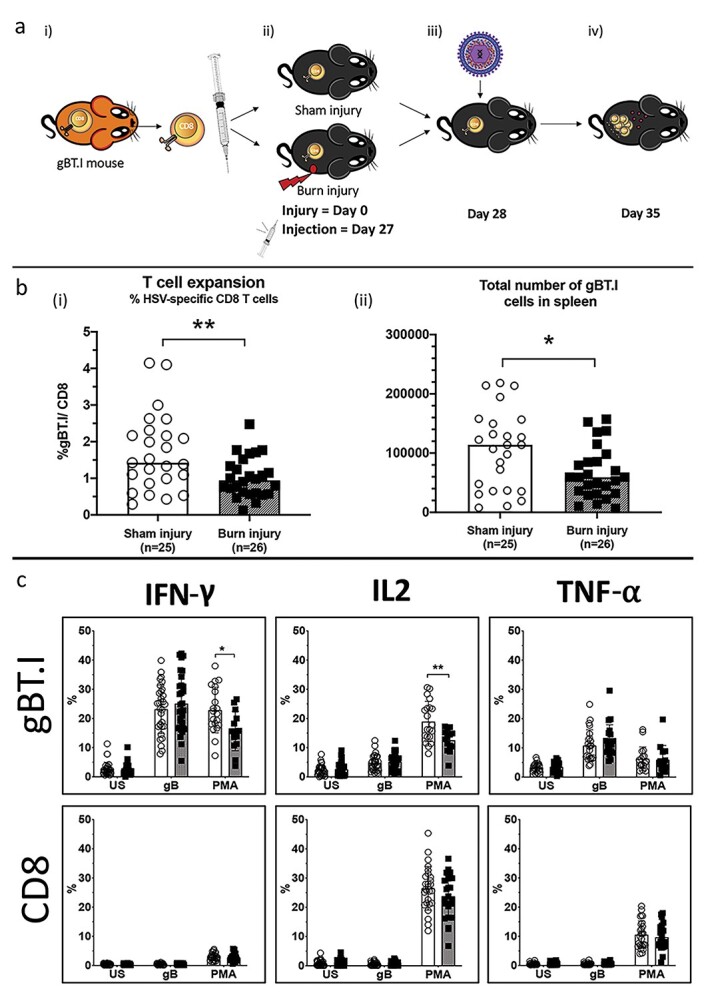
Expansion and cytokine expression of CD8^+^ T cells in response to HSV infection. (**a**) (i) Schematic diagram of the experiment. (ii) Naive T cells specific for HSV were extracted from transgenic gBT.I mice and injected into sham/burn injured wild type C57BL/6 mice 27 days post-injury. (iii) At day 28 post-injury mice were infected with HSV-1 and (iv) 7 days later mice were sacrificed and spleens were harvested for analysis. (**b**) Data shows that the proportion of (i) total CD8^+^ T cells and (ii) total number of gBT.I cells are significantly reduced in the burn injured group 7 days post-HSV infection (^**^p = 0.0054 and ^*^p = 0.0171). (**c**) Splenocytes were cultured *ex vivo* and and left unstimulated (US) or stimulated with either gB peptide or PMA. Intracellular staining was performed for IFN-γ, IL2 and TNF-α and data is shown as the percentage of CD8^+^ T cells expressing each cytokine. There was a significant reduction in the expression of IFN-γ (^*^p = 0.0212) and IL2 (^**^p = 0.0048) in gBT.I cells from the burn group. Combined data from *n* = 3 experiments. Sham injury = open circle; burn injury = closed square. *HSV* herpes simplex virus, *PMA* Phorbol 12-myristate 13-acetate - A protein kinase C activator, *IL* interleukin, *TNF-α* tumor necrosis factor alpha, *IFN-γ* interferon gamma

In light of the well-established role of CD8^+^ T cells in the epicutaneous tumour model [17], we next investigated CD8^+^ T cell function utilizing the same burn model. We used a mouse model of HSV infection that can be investigated using transgenic CD8^+^ T cells specific for the HSV-specific antigen gB (gBT.I cells) [20,24]. We determined the impact of burn injury on gBT.I cells adoptively transferred into mice at 28 days post-burn injury, which was 1 day prior to HSV infection (Figure 2). One week post-viral infection, gBT.I cells were quantified and cytokine levels (IFN-γ, IL-2, TNF-α) were determined in transgenic and endogenous CD8^+^ T cells (gating strategies for all flow cytometry analysis are shown in Figure S2, see online supplementary material). Both the proportion of CD8^+^ T cells and the total number of gBT.I cells was significantly reduced in burn injured mice at 1 week post-HSV infection when transgenic cells were transferred at 28 days post-injury, indicating impairment of antigen-specific CD8^+^ T cell expansion in response to infection (Figure 2b). Next, analysis of PMA-induced cytokine expression in *ex vivo* cultured splenocytes revealed a significant reduction in IFN-γ and IL-2 expression in gBT.I cells from burn-injured mice compared with sham-injured controls (Figure 2c). There was also a trend towards reduced IL-2 expression in endogenous CD8^+^ T cells from burned mice after PMA-stimulation (*p* = 0.142). Next, an *in vivo* cytotoxic killing assay was performed in burn injury mice with HSV infection. There was no significant difference between burn and sham, with both groups demonstrating efficient cytolytic activity against gB-pulsed target splenocytes (Figure S3, see online supplementary material). Together, these results demonstrate that the CD8^+^ T cell response to infection is significantly impacted by burn injury, culminating in reduced cell numbers and Th1 cytokine profile, whereas cytotoxic mediated cell death was unaffected.

### DCs and NK cells are not responsible for T cell dysfunction after burn injury

**Figure 3.**
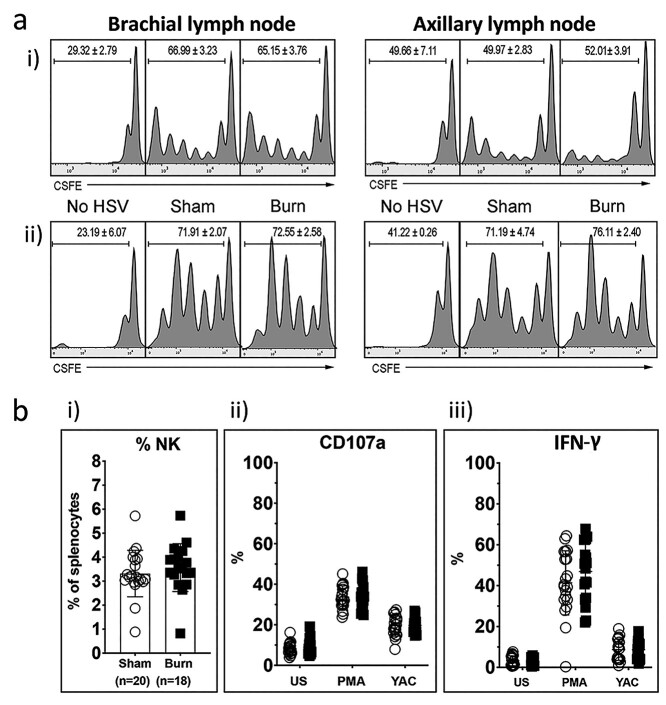
Analysis of DC and NK cells from sham- and burn-injured mice after HSV infection. (**a**) To assess antigen presentation by DCs, 7.5 × 10^5^ CFSE-labelled gBT-I cells were introduced intravenously into mice 2 (i) or 5 (ii) days post-HSV infection. Data shows T cell proliferation in the brachial lymph nodes (left) and axillary lymph nodes (right) after (i) 60 h or (ii) 42 h *in vivo*, as measured by quantification of CSFE peaks. Plots show representative data from three independent experiments. Numbers in plots indicate percent proliferated cells (mean ± SEM). (**b**) Quantification of NK cell numbers and activity after non-severe burn injury and HSV infection in splenocytes extracted from sham (open circles) or burn (closed squares) injured mice 7 days post-HSV infection. (i) Proportion of NK cells in the spleen. (ii) Expression of CD107a, which is a marker of degranulation, in unstimulated (US) NK cells, cells stimulated with PMA, and after co-culture with YAC murine cancer cells. Under the same conditions, (iii) shows the intracellular expression of IFN-γ. Gating strategies are shown in Figure S2 (see online supplementary material). Combined data from *n* = 3 experiments. *DC* dendritic cell, *NK* natural killer, *HSV* herpes simplex virus, *PMA* Phorbol 12-myristate 13-acetate - A protein kinase C activator, *IFN-γ* interferon gamma

In light of the fact that transfer of cells to burn injured mice 28 days after the injury demonstrated CD8^+^ T cell dysfunction, we next investigated whether the impact was mediated by DCs. DC antigen presentation was investigated to determine if deficiencies in antigen presentation were responsible for the observed reduction in CD8^+^ T cell expansion after viral infection. In the early stages of HSV infection (day 2), CD8α^+^ DCs are the sole DC subset responsible for antigen presentation, which occurs predominantly in the brachial lymph node [24]. During the later stages of HSV infection (day 5), CD103^+^ skin-derived DCs dominate antigen presentation to CD8^+^ T cells in the axillary lymph node [24]. To interrogate the antigen presentation capacity of these DC subsets, a Lyons–Parish assay with Carboxyluorescein succinimidyl ester - a fluorescent cell staining dye (CSFE)-labelled gBT.I cells was performed in sham/burn injured mice infected with HSV. At day 2, we observed substantial proliferation of gBT.I cells in the brachial lymph node, with small numbers of proliferating cells detectable in the axillary lymph node (Figure 3). At day 5, we observed substantial gBT.I proliferation in both the brachial and axillary lymph node. No significant difference in gBT.I cell proliferation was observed in sham or burn injured mice. This suggests antigen presentation by DCs is not impaired in this model of burn injury and viral infection.

**Figure 4.**
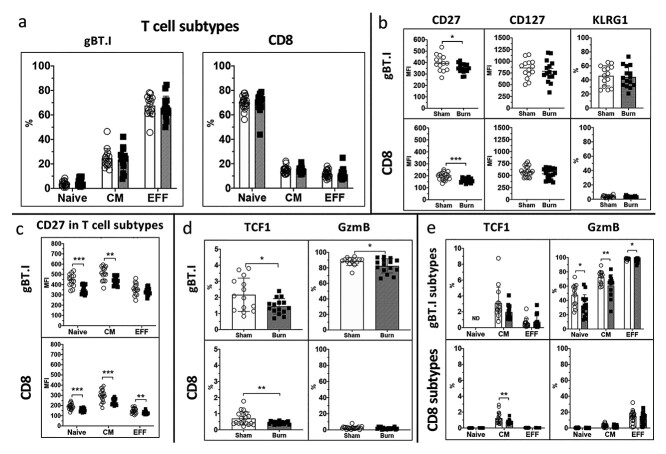
Analysis of immune parameters associated with T cell function, differentiation and memory in CD8^+^ T cells after burn injury and HSV infection. Splenocytes were harvested from sham (open circles) or burn (closed squared) injured mice at day 7 post-HSV infection and endogenous CD8^+^ T and gBT.I cells were assessed for surface and intracellular expression of various immune parameters. Gating strategy is shown in Figure S2 (see online supplementary material). (**a**) The proportion of T cell subtypes in gBT.I cells and endogenous CD8^+^ T cells (expressed as % of cells ± SD). Subtypes were classified as naive (CD44-CD62L+), central memory (CM, CD44 + CD62L+) and effector cells (EFF, CD44 + CD62L-). (**b**) Expression of surface markers CD27, CD127 and KLRG1 in gBT.I and endogenous CD8^+^ T cells. CD27 and CD127 expression is shown as median fluorescent intensity (MFI), while KLRG1 expression is represented as percentage positive T cells. Data identified significant changes in the expression of CD27 in both endogenous CD8^+^ T (^***^p = 0.0002) and gBT.I (^*^p = 0.0453) cells. (**c**) To interrogate this further, the expression of these markers was also quantified in T cell subsets. (**d**) Shows the expression of intracellular markers TCF1 and GzmB in gBT.I and endogenous CD8^+^ T cells, shown as % positive cells. Data identified significant changes in the expression of TCF1 in both gBT.I (^*^p = 0.0280) and endogenous CD8^+^ T cells (^**^p = 0.0087), and GzmB in gBT.I cells (^*^p = 0.0454). (**e**) To analyse this further, the expression of these markers was also quantified in T cell subsets. Each graph shows combined data from *n* = 4 independent experiments. *HSV* herpes simplex virus

**Figure 5.**
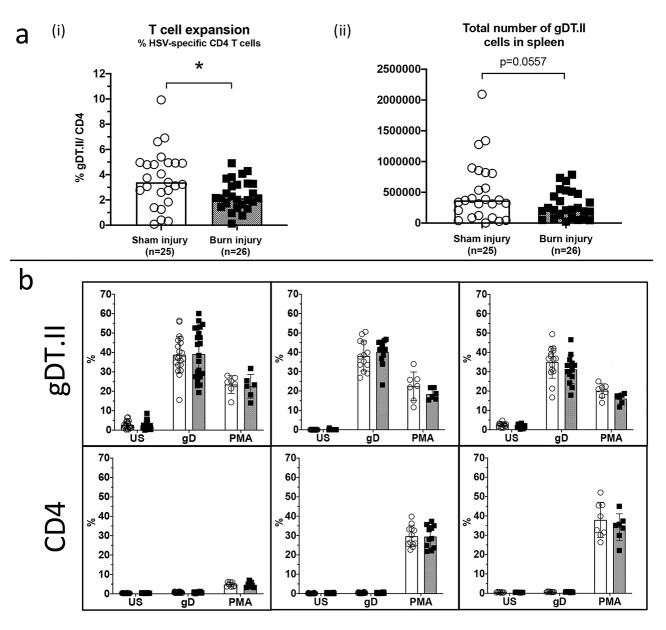
Expansion and cytokine expression of CD4^+^ T cells in response to HSV infection. Experimental schematic is shown in Figure 2a, but this experiment used gDT.II mice instead of gBT.I. (**a**) Data shows that the proportion of gDT.II cells of total CD4^+^ T cells is significantly reduced in the burn injured group 7 days post-HSV infection (^*^p = 0.0137), and this is reflected by a trend towards less total gDT.II cells at harvest (*p* = 0.1675) (ii). (**b**) Splenocytes were cultured *ex vivo* and left unstimulated (US) or stimulated with either gD peptide or PMA. Intracellular staining was performed for IFN-γ, IL2 and TNF-α and data is shown as the percentage of T cells expressing each cytokine. Combined data from *n* = 3 experiments. Sham injury = open circle, burn injury = closed square. *HSV* herpes simplex virus, *PMA* Phorbol 12-myristate 13-acetate - A protein kinase C activator, *IL* interleukin, *TNF-α* tumor necrosis factor alpha, *IFN-γ* interferon gamma

As CD8^+^T cell responses are often co-ordinated with innate NK cell immune responses, we also investigated the impact of burn injury on NK cell numbers, cytokine expression and activity. Splenocytes were extracted from burn injured mice 7-days post HSV infection and plated unstimulated, stimulated with PMA or cultured with YAC murine cancer cells, and NK cell activity was determined. NK cell numbers, degranulation and cytokine expression were not significantly different in burn injured mice (Figure 3), suggesting the burn did not impact the innate NK cell response to viral infection.

### Burn injury significantly impacts the differentiation and memory formation of T cells in response to HSV infection

We investigated whether the reduction in gBT.I cells observed in burn mice may be due to altered secondary CD8^+^ T cell priming signals leading to dysfunction in cell differentiation. CD8^+^ T cells post-burn and HSV infection were analysed for T cell differentiation and memory formation markers (Figure 4). Endogenous CD8^+^ T cells and transgenic gBT.I cells were classified as naive (T_naive_ CD62L^+^CD44^−^), central memory (T_CM_, CD62L^+^CD44^+^) and effector (T_EFF_, CD62L^−^CD44^+^), which includes both cytotoxic effectors and effector memory cells (Figure 4a). There was no detectable difference in the proportion of T_naive_, T_CM_ or T_EFF_ cells in the endogenous CD8^+^ T cells. However, there was a trend towards an increased proportion of T_naive_ cells in the gBT.I population (*p* = 0.112). Next, we analysed the expression of markers associated with T cell differentiation and memory formation (CD27, CD127, KLRG1) post-burn and viral infection. A significant reduction in CD27 expression was observed in both the transgenic gBT.I and endogenous CD8^+^ T cell populations (Figure 4b). To investigate further, we assessed the expression of CD27, CD127 and KLRG1 markers in the endogenous CD8^+^ T_naive_, T_CM_ and T_EFF_ cells and found that CD27 was significantly reduced in all subsets (Figure 4c). Similarly there was reduced expression of CD27 on T_naive_, and T_CM_ gBT.I cells. Next, to determine changes in effector function and T cell exhaustion, we quantified intracellular markers GzmB and TCF1, respectively (Figure 4d). In the burn-injured group there was a significant reduction in TCF1 expression in both endogenous CD8^+^ T cells and gBT.I cells at day 7 post-infection, and a significant reduction in GzmB expression in gBT.I cells. Analysis of these markers in CD8^+^ T_naive_, T_CM_ and T_EFF_ cells showed that TCF1 was significantly reduced in endogenous T_CM_ cells, and that GzmB expression was significantly reduced in all gBT.I cell subtypes after burn injury and viral infection (Figure 4e). The observed changes in CD27, GzmB and TCF1 indicate that T cell differentiation and/or memory potential may be impaired after burn injury.

### Antigen-specific CD4 ^+^ T cell expansion and cytokine production is also impaired in the response to HSV infection after burn injury

CD8^+^ T cell responses can be modulated by CD4^+^ T helper cells, so we investigated whether burn injury impacted the numbers, cytokine expression or activity of these cells after infection. To investigate this, transgenic CD4^+^ T cells specific for the HSV antigen gD (gDT.II cells) were adoptively transferred into sham or burn injured mice the day prior to HSV infection 28 days post-injury. Spleen cells were extracted from burn injured mice 7-days post HSV infection and plated with MuTu DCs +/− gD peptide for analysis of CD4^+^ T cell activity. gDT.II cell numbers were significantly reduced in the spleens of burn injured mice 7 days post-infection (Figure 5). This data shows that in addition to CD8^+^ T cell dysfunction, CD4^+^ T cell responses to infection are also significantly impaired after burn injury.

## Discussion

Burn injury and immune suppression are intrinsically linked, however studies on the mechanisms, persistence and prevalence of this dysfunction, especially in relation to non-severe burn injuries, are limited. Redefining burn injury as a chronic disease, recognising the long-term health impacts associated with non-severe burns and defining the mechanisms that underpin these health impacts has the potential to profoundly impact patient treatment.

In this work we show that non-severe burn injury in mice increases cancer incidence and causes significant and persistent immune dysfunction, characterized by inefficient T cell responses in the context of infection, resulting in significantly reduced numbers of T cells at the peak of the immune response to HSV. Analysis of immune parameters associated with CD8^+^ T cell function, differentiation and memory formation also showed that these processes are impaired. Assessment of antigen presentation during the immune response to HSV demonstrated that CD8^+^ T cell activation by DCs was not impaired in this model, and NK cell activity was also unaffected. Importantly, the defects in T cell function were observed despite the transgenic T cells being introduced into burn injured animals after recovery from their burn. This suggests that a systemic mediator of this dysfunction exists at the time of T cell transfer, but this is yet to be identified.

Despite showing significant reductions in CD8^+^ T cell numbers in our model, the capacity of these cells for cytotoxic mediated cell death was not affected. While this may seem counterintuitive, it is not necessarily unexpected. The link between burn injury and secondary pathologies, including increased incidence and severity of infections, was only apparent after large-scale longitudinal population studies, which is indicative of subtle but persistent effects on immunity. Analysis of cytokine expression in CD8^+^ T cells introduced post-burn indicated significant reductions in pro-inflammatory effector cytokine expression potential in gBT.I cells 1 week post-infection. IFN-γ is an important Th1 effector cytokine that modulates T cell activation, differentiation and function, and in the context of infection IFN-γ acts to block viral replication (reviewed in [25]). IL-2 drives proliferation and differentiation of T cells and is important in T memory cell formation and survival [26]. We also observed a reduction in GzmB expression from gBT.I cells, a serine protease important for CTL activity in effector cells. Resting CD8^+^ T_naive_ and T_CM_ cells have no cytolytic granules containing GzmB, however they start expressing GzmB a few days after antigen encounter [27]. For this to occur, T cells must receive a third signal cytokine, which is provided by IL-12 or type 1 interferons [28]. This signal also increases clonal expansion of CD8^+^ T cells by increasing production of Bcl-3, a transcription factor that promotes cell survival. Our results suggest that the timing and/or magnitude of this signalling may be impacted by burn injury. Taken together, the significant reductions observed in IFN-γ, IL-2 and GzmB expression from gBT.I cells indicates potential defects in signalling, with consequences for T cell expansion, survival, differentiation and memory formation.

T cell memory formation is crucial to the development of long-lasting immunity to pathogens and cancer prevention [29–31]. This process requires the activation and differentiation of T cells, evidenced by expression of markers associated with memory formation and longevity. After expansion, 90–95% of T cells undergo apoptosis and the surviving cells become long-lasting memory cells [32]. Here we show that CD8^+^ T cells from burn injured mice have significantly reduced expression of CD27 and TCF1 1 week post-viral infection. CD27 is a co-stimulatory immune checkpoint marker that plays a crucial role in the maintenance and survival of T cells during activation and is required for the generation and long-term maintenance of T cell immunity and memory [33,34]. The ligand for CD27 is CD70, which is expressed on antigen presenting cells in early immune responses [35] and on CD4^+^ Th1 cells in later immune cell responses [36]. In addition, CD27 has also been reported to stimulate IL-2 production for CD8^+^ T cell proliferation [37]. TCF1 is a transcription factor that is critical for CD8^+^ T cell development, differentiation, memory maintenance and recall responses [38]. Therefore, reduced expression of IFN-γ and CD27 may indicate a reduction in Th1-mediated CD8^+^ T cell responses, and CD27 and TCF1 levels in CD8^+^ T cells indicate an overall reduction in memory T cell potential after burn.

Reduced T memory formation post-burn injury is expected to have cumulative effects on immune function and disease susceptibility, leaving patients vulnerable to repeat infections, as observed in epidemiological studies [6]. Additionally, our observations indicate a potential mechanism for the observed increase in cancer post-burn. A recent study demonstrated that tissue-resident memory T cells (TRM) play a crucial role in the melanoma surveillance that suppresses tumour growth in tumour-free mice in this model [17]. Future work to characterize the impact of burn injury on memory T cells, tissue-resident memory T cell formation and localisation, and tumour-specific T cell responses using techniques such as RNA-sequencing and T cell receptor-sequencing alongside other functional and interventional studies, will provide more insight into T cell dysfunction and mechanisms resulting in increased secondary morbidity post-burn injury.

Recent studies have shown that CD4^+^ T cells can enhance CD8^+^ T cell responses during priming through interactions with XCR1^+^DCs. Licensing of DCs by CD4^+^ T helper cells upregulates IL-12 and CD70L, leading to optimal CD8^+^ T cell effector differentiation via CD27 costimulation [39]. CD4^+^ T cell help has also been shown to be important for optimal CD8^+^ T cell memory formation [40]. Our results demonstrated reductions in CD4^+^ T cell expansion in response to infection, which we hypothesize contributes to CD8^+^ T cell dysfunction. The role of CD4^+^ T cells in CD8^+^ T cell dysfunction post-burn will be a focus of future research.

## Conclusions

In this study we have demonstrated that non-severe burn injury has long-lasting impacts on immune function, characterized by inefficient T cell responses. This study is the first to show significant differences in cancer susceptibility and antigen-specific T cell responses in the context of a healed non-severe burn injury. However, longer time points will be required to determine the longevity of the responses reported in our study, whilst future work will focus on identifying the underlying mechanisms that lead to this immune dysfunction. This will provide an opportunity for better clinical intervention to decrease the incidence of long-term secondary morbidities associated with immune dysfunction after burn injury.

**Abbreviations**
DC: Dendritic cell; GzmB: Granzyme B; HSV: Herpes simplex virus; ICS: Intracellular staining; NK: Natural killer cell; TBSA: Total body surface area; TCF1: T cell transcription factor 1.

## Supplementary Material

Figure_S1_tkac016

Figure_S2_tkac016

Figure_S3_tkac016
