# Supplementary figures and images for: Non-severe burn injury increases cancer incidence in mice and has long-term impacts on the activation and function of T cells
Source: Burns Trauma. 2022 Apr 29;10:tkac016. doi: 10.1093/burnst/tkac016 (PMC9054911; doi:10.1093/burnst/tkac016)

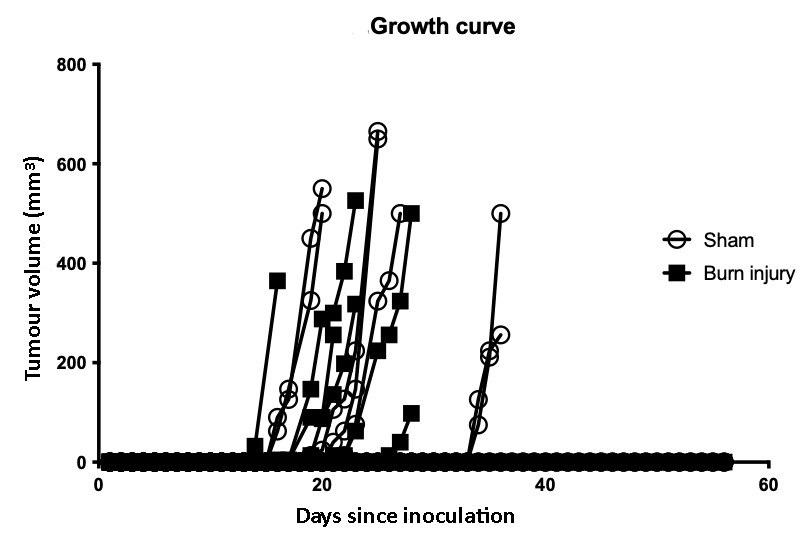

Supplement: Figure_S1_tkac016 [file figure_s1_tkac016.jpeg]

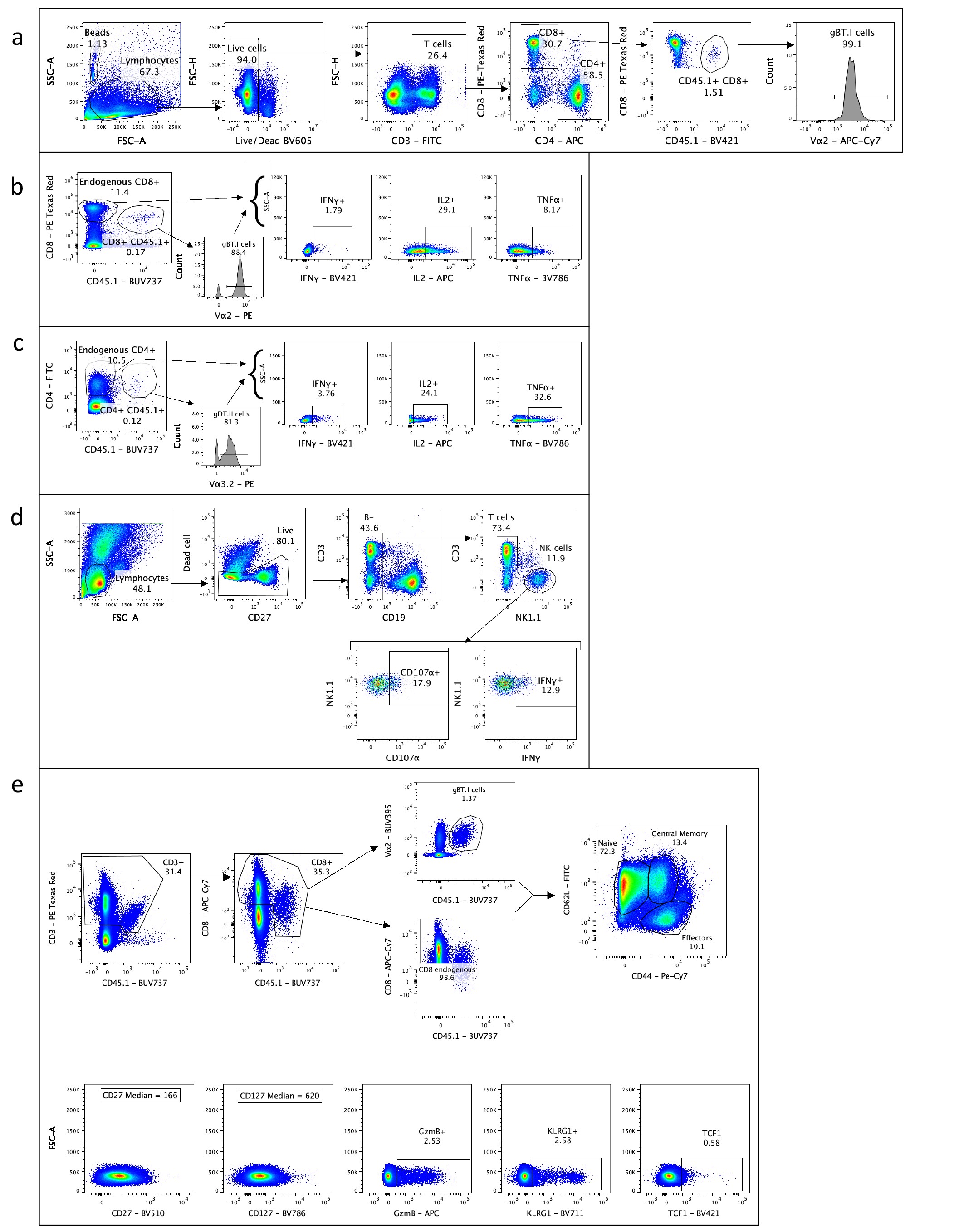

Supplement: Figure_S2_tkac016 [file figure_s2_tkac016.jpeg]

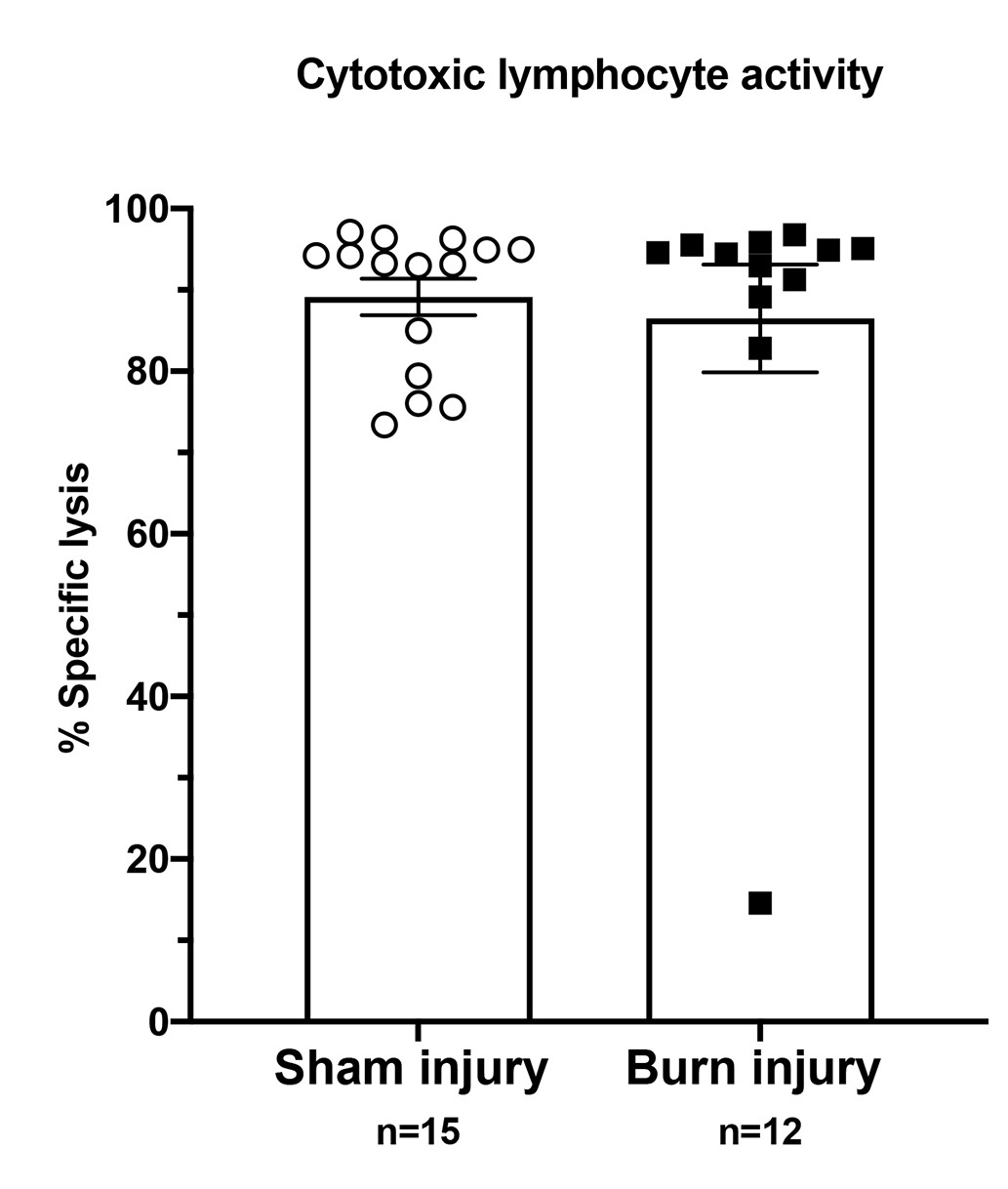

Supplement: Figure_S3_tkac016 [file figure_s3_tkac016.jpeg]
